# The association between team behaviors and competitive anxiety among team-handball players: the mediating role of achievement goals

**DOI:** 10.3389/fpsyg.2024.1417562

**Published:** 2024-06-21

**Authors:** Xiaolin Wang, Zhuo Sun, Lei Yuan, Depeng Dong, Delong Dong

**Affiliations:** ^1^Department of Physical Education, Ludong University, Yantai, China; ^2^School of Philosophy and Sociology, Jilin University, Changchun, China; ^3^School of Physical Education and Sport Science, Qufu Normal University, Qufu, China

**Keywords:** team behavior, controlling coaching behavior, team cohesion, achievement goals, competitive anxiety

## Abstract

Team sports athletes may encounter significant stress, leading to competitive anxiety. The anxiety levels can be influenced by team behaviors and achievement goals. This study aims to investigate the relationship between team behaviors (i.e., perceptions of controlling coaching behavior and team cohesion) and competitive anxiety, and to examine the mediation effects of achievement goals (i.e., task-oriented and ego-oriented) on the relationship. A total of 298 team-handball players were involved in the study, ages ranging from 16 to 24 years old (*M* = 18.44, SD = 3.09). A cross-sectional research design was adopted, and structural equation modeling was utilized to analyze path coefficients and mediating effects. Findings indicated that perceptions of controlling coaching behaviors had significant positive predictions for state and somatic anxiety (*β* = 0.22, 0.29) and negative predictions for self-confidence (*β* = −0.19). Team cohesion had significant negative predictions for state anxiety (*β* = −0.31) and positive predictions for self-confidence (*β* = 0.58). In addition, ego-oriented goals play a positive mediating role in the relationship between team behaviors and competitive anxiety (*β* = 0.03–0.35), while task-oriented goals play a negative mediating role in the relationship between team behaviors and competitive anxiety (β = −0.18 - −0.03). In conclusion, team behaviors have a significant relationship with competitive anxiety, with achievement goals playing a mediating role among them. Therefore, to alleviate team sports athletes’ competitive anxiety, it is recommended to reduce coach control behaviors, enhance team cohesion, and employ psychological training methods (e.g., mindfulness or meditation) to strengthen athletes’ task-oriented goals.

## Introduction

In competitive sports, athletes often encounter various psychological pressures, arising from the competition itself, expectations from coaches and the team, the scrutiny of the audience and the public, as well as the stress associated with injuries and health concerns, both before and during competitions. These potential pressures and threats can result in competitive anxiety, ultimately affecting their athletic performance and mental well-being (Craft et al., 2003; Woodman and Hardy, 2003).

In this field, cognitive motivational relational theory by Lazarus (2000) emphasizes the importance of cognition and goals in generating emotions, providing a potentially fruitful theoretical framework for investigating anxiety within competitive contexts. The theory posits that goals act as crucial mediators between cognitive appraisal (appraising the situation or behaviors) and emotional responses (e.g., anxiety) (Lazarus, 2000; Uphill and Jones, 2007). For example, during competitions, athletes’ performance can be influenced by how they interpret their coach’s feedback. If team sports athletes perceive the coach’s reprimand as hindering their goal of winning, it may induce feelings of discouragement and anxiety, impacting their performance negatively. However, if they view the feedback as constructive criticism aimed at improvement, it can motivate them to make necessary adjustments and strive for better results.

In team sports, team behaviors (i.e., the behaviors of coaches and teammates) significantly influence the emotional states of athletes, particularly competitive anxiety. Coaching behaviors, especially controlling ones, may cause athletes to excessively focus on the game’s outcome, creating a threatening environment (e.g., fear of failure and lack of recognition), thus increasing competitive anxiety (Ramis et al., 2017). Additionally, teammate behaviors, such as team cohesion, play a crucial role in helping athletes cultivate a more positive mindset (Craft et al., 2003). This contributes to boosting the self-confidence of athletes, enabling them to more effectively cope with competitive pressure and, as a result, alleviate competitive anxiety (Craft et al., 2003; Oh and Gill, 2017). Based on current empirical research, the majority of studies confirm that coach controlling behaviors positively predict athletes’ competitive anxiety (Ramis et al., 2017; Cho et al., 2019), while team cohesion negatively predicts competitive anxiety (Oh and Gill, 2017; Najafi et al., 2018). However, there are still some studies indicating a lack of significant correlation between coach controlling behaviors and competitive anxiety (Vealey et al., 1998; Madson, 2021), as well as between team cohesion and competitive anxiety (Anderson, 2015; Haddera, 2015). Therefore, one of the purposes of this research was to further examine the relationship between team behaviors and competitive anxiety.

According to the achievement goal theory (Dweck, 1986), individual achievement goals can be divided into task-oriented and ego-oriented. Task-oriented athletes focus on self-learning and skill improvement, and this learning goal orientation helps alleviate competitive anxiety. Conversely, athletes holding primarily ego-oriented motivation excessively focus on the outcome of the competition, thereby increasing the likelihood of competitive anxiety. Furthermore, team behavior appears to be capable of altering athletes’ competitive anxiety states by influencing their goal orientation. The controlling style of coaching behavior can reinforce ego-oriented goals in athletes through excessive supervision, guidance, and decision-making (Syrmpas and Bekiari, 2018), potentially leading to an increased level of competitive anxiety. Conversely, team cohesion, which emphasizes cooperation and shared goals, helps foster the task orientation of athletes, reducing excessive focus on competition outcomes and alleviating competitive anxiety (Prapavessis and Carron, 1996). Therefore, based on achievement goal theory and cognitive motivational relational theory, achievement goals mediate the relationship between team behavior (e.g., perceptions of controlling coaching behavior and team cohesion) and competitive anxiety. Current empirical research indicated a negative correlation between ego-oriented goals and positive coaching behavior or team cohesion, while a positive correlation existed between task-oriented goals and positive coaching behavior or team cohesion among team-handball players (Balaguer et al., 2002; Horn et al., 2012). Furthermore, ego-oriented goals were positively associated with competitive anxiety, whereas task-oriented goals showed a negative correlation with competitive anxiety among team-handball players (Abrahamsen et al., 2008; Duica et al., 2014). Despite theoretical and practical indications that achievement goals might mediate between team behavior and physical activity, there is a lack of comprehensive research investigating the mediating role of achievement goals in the relationship between team behavior and competitive anxiety. Hence, another objective of this study was to examine the mediating effect of achievement goals on the relationship between team behavior and competitive anxiety.

In summary, existing research indicates an inconsistent relationship between team behavior and competitive anxiety, with no conclusive evidence indicating that achievement goals play a mediating role between team behavior and competitive anxiety. Therefore, the primary aim of this study is to examine the correlation between team behavior (i.e., perceptions of controlling coaching behavior and team cohesion), achievement goals (i.e., task-oriented and ego-oriented), and competitive anxiety. Furthermore, the study aims to examine the potential mediating effect of achievement goals on the relationship between team behavior and competitive anxiety.

## Materials and methods

### Participants

A total of 298 team-handball players (76 males and 222 females) from three provincial clubs in the Shandong, Jiangsu, and Beijing regions of China participated in the study. Employing a randomized approach, three clubs were randomly selected from the pool of teams participating in each regional league. The sample size of 298 participants meets the minimum required sample size for structural equation modeling analysis, which was calculated as 119 using G-Power (*α* = 0.05, power = 0.95). The participants were categorized into three age groups: U17 (15 to <17 years), U19 (17 to <19 years), and Senior group (≥ 19 years). Informed consent was obtained from all participants or their legal guardians if under 18 years of age. Regarding training experience, over 89.3% of participants reported having three or more years of training experience. Additionally, the participants’ sports levels ranged from amateur to elite. Specific characteristic details can be found in Table 1.

**Table 1:** General characteristics of the participants (*n* = 298).

Characteristics	Category	Frequency	Percentage
Gender	Male	76	25.5%
	Female	222	74.5%
Age group	U17	109	36.6%
	U19	119	39.9%
	Senior	70	23.5%
Training experience	1–2 years	32	10.7%
	3–4 years	157	52.7%
	≥ 5 years	109	36.6%
Sports level	Amateur	114	38.3%
	Sub-elite	114	38.3%
	Elite	70	23.4%

U17, 15 to < 17 years; U19, 17 to < 19 years; Senior, ≥ 19 years.

#### Measures

In this research, the respondents were requested to fill out a questionnaire that included demographic information (sex, age, training experience and sports level), Controlling Coach Behaviors Scale (CCBS) (Bartholomew et al., 2010), Group Environment Questionnaire (GEQ) (Ma, 2004), Task and Ego Orientation in Sports Questionnaire (TEOSQ) (Whitehead and Duda, 1998), and Competitive State Anxiety Inventory-2 (CSAI-2) (Martens et al., 1990). To ensure internal validity, the scales were adapted and validated within the Chinese cultural context, incorporating feedback from an expert panel, and conducting internal consistency checks concurrently (Cronbach’s alpha values >0.7) (Diotaiuti et al., 2017; Taber, 2018).

#### Controlling coaching behaviors

The CCBS (Bartholomew et al., 2010) was used to measure team-handball players’ perceptions of controlling coaching behaviors. The CCBS scale encompasses four subscales with a total of 15 items: controlling use of rewards (e.g., “the only reason my coach rewards/praises me is to make me train harder”), negative conditional regard (e.g., “my coach pays me less attention if I have displeased him/her”), excessive personal control (e.g., “my coach tries to control what I do during my free time), and judging and devaluing (e.g., “my coach is very judgmental if I am not competing well”). A 5-point Likert scale, scored from 1 to 5, was used for responses. Higher scores indicate a stronger perception of controlling coaching behaviors. In this study, the internal consistency results for the four subscales indicate an acceptable reliability, with Cronbach’s alpha values ranging from 0.77 to 0.91.

#### Team cohesion

The GEQ (Harter and Connell, 1984) was used to measure team cohesion among team-handball athletes. Ma (2004) adapted the GEQ based on the cultural attributes of Chinese team-handball athletes. This adapted questionnaire consists of four dimensions (15 items): group task attraction (e.g., “I’m pleased with the competitive drive in our team”), group social attraction (e.g., “I’m willing to participate in the team’s social activities”), group task integration (e.g., “Our team is united in striving to achieve our goals.”), and group social integration (e.g., “Our team members often gather together for social events”). The internal consistency results for the four subscales demonstrate an acceptable reliability in this sample (Cronbach’s alpha, α = 0.68–0.91).

#### Achievement goals

The TEOSQ (Whitehead and Duda, 1998) is a widely utilized instrument in the field of sports to evaluate athletes’ achievement goals, specifically in terms of their task orientation and ego orientation. In this study, this scale was used to measure athletes’ achievement motivation. The TEOSQ questionnaire comprises a total of 13 items, with 7 items designed to measure task orientation (e.g., motivation focused on self-improvement, personal mastery of skills, and achieving specific goals) and an additional 6 items intended to assess ego orientation (e.g., motivation driven by the pursuit of victory, seeking recognition, and competing with others). The internal consistency results for this scale demonstrate acceptable reliability (Cronbach’s alpha, *α* = 0.89–0.93).

#### Competitive anxiety

The CSAI-2 (Martens et al., 1990) was used to measure the competitive anxiety levels of team-handball athletes. Zhu (1993) adapted the CSAI-2 based on the characteristics of Chinese athletes and demonstrated the scale’s strong reliability and validity. This adapted scale is comprised of three subscales: state anxiety (e.g., “I’m worried about this upcoming match”), somatic anxiety (e.g., “I feel tense physically”), and self-confidence (e.g., “I’m confident about this match”), with each subscale containing 9 items, making a total of 27 items. In this study, the internal consistency results for three dimensions indicate an acceptable reliability (Cronbach’s alpha, *α* = 0.88–0.93).

#### Procedures and research design

Permission for the athlete’s survey was obtained from the Provincial Sports Bureau and the coaches. Before conducting this survey, all respondents were informed about the purpose of the study. All responses were anonymous, and participation was voluntary. Data was collected in September 2023. All structured questionnaires were distributed to the team-handball payers before a training session and collected on-site 20 min later, ensuring that no external interference occurred during that time to avoid any bias or influence. Among the 307 athletes invited to participate in this survey, 9 did not complete the test (the response rate was 97.1%). This survey followed the guidelines outlined in the Declaration of Helsinki (World Medical Association, 2013). The ethical approval and consent procedures for this study were approved by the Ludong University Ethics Committee (LDU-IRB202311002).

### Statistical analysis

Descriptive statistics and correlations analysis were performed by using Statistical Package of the Social Sciences (IBM SPSS Statistics, version 25.0). Demographic characteristics, perceptions of controlling coaching behaviors, team cohesion, achievement goals, and competitive anxiety underwent descriptive statistics analysis, including the calculation of means, standard deviations, and percentages. Structural equation modeling (SEM) was conducted using Analysis of Moment Structures (AMOS, version 24.0). Confirmatory factor analysis (CFA) was utilized to assess the fitness of the model for each construct. The bootstrap method was used for mediation effect analysis (Parameters: bootstrap samples, 2000; PC confidence intervals, 95; BC confidence intervals, 95). To evaluate the overall model fit, the following fit indices were considered: Comparative Fit Index (CFI), Tucker–Lewis Index (TLI), Goodness of Fit Index (GFI), Normed Fit Index (NFI), Root Mean-Square Error of Approximation (RMSEA), and the likelihood ratio (χ2/df). The following criteria indicated an adequate model fit: RMSEA ≤0.08, χ2/df < 5, and other indices (CFI, TLI, GFI, and NFI) ≥ 0.9 (Heck and Thomas, 2020; Kline, 2023).

## Results

### Descriptive statistics

Table 2 shows the means, standard deviations, and correlations among competitive anxiety (i.e., somatic anxiety, state anxiety, and confidence), achievement goals, team cohesion, and perceptions of controlling coaching behaviors. Notably, perceptions of controlling coaching behaviors were positively associated with state anxiety, somatic anxiety, and task-oriented motivation, while exhibiting a negative correlation with self-confidence. Additionally, team cohesion was positively associated with self-confidence, task-oriented goals, and ego-oriented goals, while negatively associated with somatic anxiety. Furthermore, both task-oriented and ego-oriented goals displayed positive associations with self-confidence. In the realm of competitive anxiety, self-confidence exhibited a negative relationship with both somatic anxiety and state anxiety.

**Table 2:** Means, standard deviations, and correlations.

Variables	1	2	3	4	5	6	7
1. STA	1						
2. SOA	0.96^***^	1.00					
3. Confidence	−0.21^**^	−0.20^**^	1				
4. TOG	0.03	−0.07	0.27^***^	1			
5. EOG	0.05	−0.05	0.34^***^	0.73^***^	1		
6. PCCB	0.23^***^	0.26^***^	−0.16^*^	0.29^***^	0.04	1	
7. TC	−0.09	−0.15^*^	0.39^***^	0.54^***^	0.81^***^	−0.08	1
Mean	2.52	2.18	2.75	4.19	3.80	2.61	4.08
SD	0.73	0.68	0.60	0.67	0.82	0.87	0.58

EOG, ego-oriented goal; PCCB, perceptions of controlling coaching behavior; SOA, somatic anxiety; STA, state anxiety; TC, team cohesion; TOG, task-oriented goal. Confidence, SOA and STA are the subscales of competitive anxiety. ^*^p < 0.05, ^**^p < 0.01, ^***^p < 0.001.

### Structural equation modeling results

**Figure 1:**
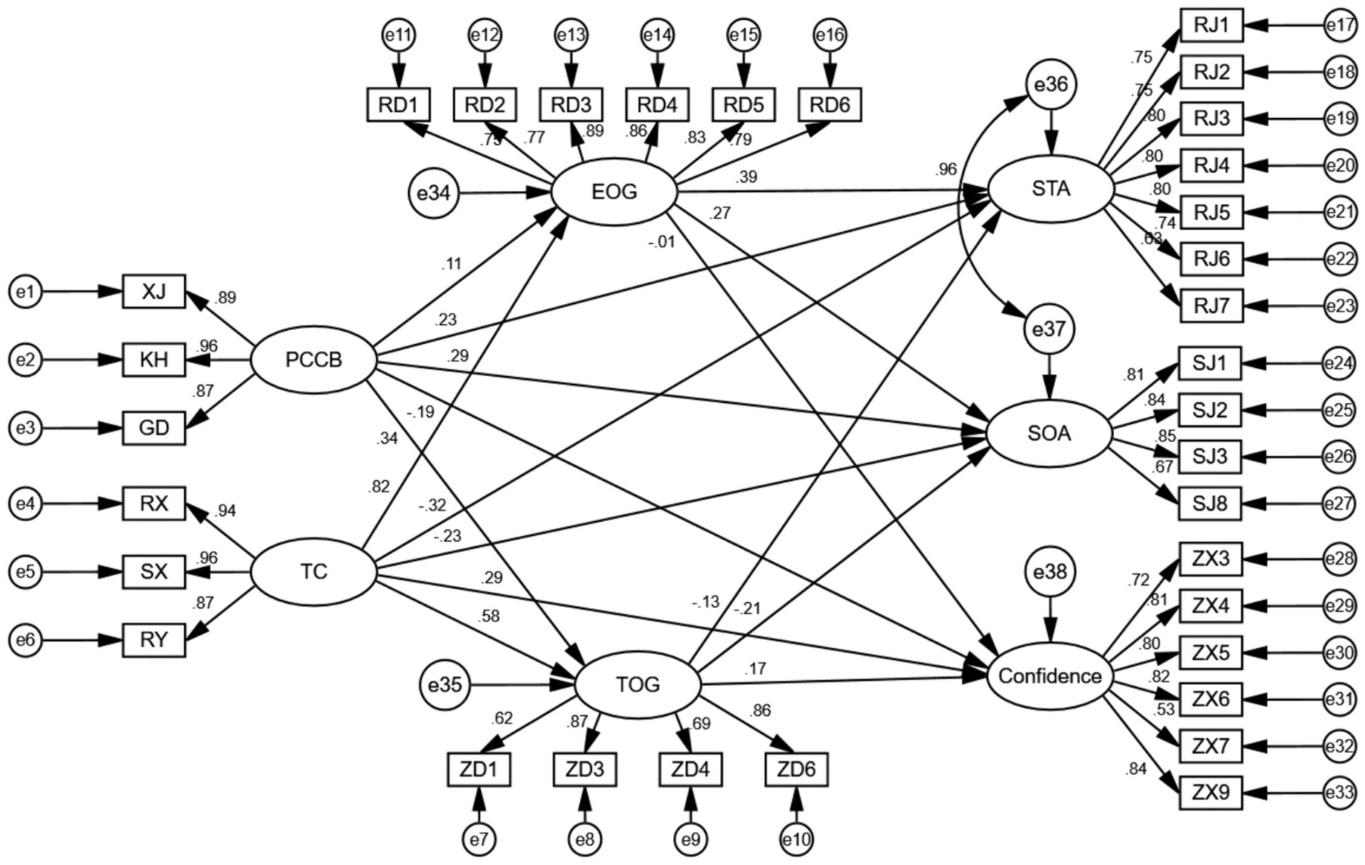
Structural equation modeling results. Note: EOG, ego-oriented goal; PCCB, perceptions of controlling coaching behavior; SOA, somatic anxiety; STA, state anxiety; TOG, task-oriented goal. This model has an acceptable fit (χ^2^/*df* = 2.912; CFI = 0.921; TLI = 0.907; IFI = 0.922; GFI = 0.831). With the exception of the paths EOG → Confidence, TOG → STA, and TC → SOA, all other paths were significant at p < 0.05 level.

The structural equation modeling results are shown in Figure 1. In this structural model, perceptions of controlling coaching behaviors exhibited significant positive predictions for task-oriented and ego-oriented goals, state anxiety, and somatic anxiety (*β* = 0.35, 0.10, 0.22, 0.29, *p* < 0.05), while concurrently yielding negative predictions for self-confidence (*β* = −0.19, *p* < 0.05). Team cohesion emerged as a robust predictor, demonstrating significant positive associations with task-oriented and ego-oriented goals, as well as self-confidence (*β* = 0.58, 0.82, 0.28, *p* < 0.05), while showing a negative prediction for state anxiety (*β* = −0.31, *p* < 0.05). Moreover, the task-oriented goal displayed a significant positive prediction for self-confidence (*β* = 0.17, *p* < 0.05), and a simultaneous negative prediction for somatic anxiety (*β* = −0.20, *p* < 0.05). Additionally, the ego-oriented goal showed a significant positive prediction for state anxiety and somatic anxiety (*β* = 0.38, 0.24, *p* < 0.05).

Table 3 illustrates the Bootstrap mediation effects of achievement goals (i.e., task-oriented and ego-oriented) serving as mediators in the relationship between coaching behaviors and competitive anxiety, as well as in the relationship between team cohesion and competitive anxiety. The findings indicated that ego-oriented goals mediated between perceptions of controlling coaching behavior and state anxiety, as well as between perceptions of controlling coaching behavior and somatic anxiety. Additionally, ego-oriented goals served as a mediator between team cohesion and both state anxiety and somatic anxiety. However, there was no mediating effect of ego-oriented goal observed between perceptions of controlling coaching behaviors and self-confidence, nor between team cohesion and self-confidence. On the other hand, the mediating role of task-oriented goals was evident in the relationship between perceptions of controlling coaching behavior and somatic anxiety.

**Table 3:** Bootstrap mediation effects of achievement goals in the relationship between team cohesion and competitive anxiety, as well as perceptions of controlling coaching behavior and competitive anxiety.

Effect	Path	Beta		95% CI	
			*P*	Lower	Upper
Direct effect	PCCB → STA	0.161	^**^	0.052	0.293
Indirect effect	PCCB → EOG → STA	0.031	^**^	0.006	0.077
Indirect effect	PCCB → TOG → STA	−0.03	0.312	−0.094	0.031
Total effect	PCCB → STA	0.162	^**^	0.064	0.275
Direct effect	PCCB → SOA	0.273	^***^	0.142	0.440
Indirect effect	PCCB → EOG → SOA	0.029	^*^	0.001	0.080
Indirect effect	PCCB → TOG → SOA	−0.068	^*^	−0.149	−0.001
Total effect	PCCB → SOA	0.233	^***^	0.114	0.385
Direct effect	PCCB → SC	−0.148	^*^	−0.267	−0.030
Indirect effect	PCCB → EOG → SC	−0.001	0.945	−0.029	0.027
Indirect effect	PCCB → TOG → SC	0.047	0.177	−0.025	0.119
Total effect	PCCB → SC	−0.102	^*^	−0.206	−0.002
Direct effect	TC → STA	−0.347	^*^	−0.607	−0.049
Indirect effect	TC → EOG → STA	0.351	^*^	0.082	0.622
Indirect effect	TC → TOG → STA	−0.080	0.323	−0.246	0.087
Total effect	TC → STA	−0.076	0.375	−0.251	0.072
Direct effect	TC → SOA	−0.333	0.114	−0.688	0.091
Indirect effect	TC → EOG → SOA	0.323	*	0.000	0.654
Indirect effect	TC → TOG → SOA	−0.181	0.072	−0.366	0.016
Total effect	TC → SOA	−0.191	0.080	−0.428	0.027
Direct effect	TC → SC	0.359	^*^	0.057	0.706
Indirect effect	TC → EOG → SC	−0.008	0.967	−0.314	0.286
Indirect effect	TC → TOG → SC	0.125	0.185	−0.070	0.313
Total effect	TC → SC	0.476	^***^	0.336	0.636

EOG, ego-oriented goal; PCCB, perceptions of controlling coaching behavior; SOA, somatic anxiety; SC, self-confidence; STA, state anxiety; TC, team cohesion; TOG, task-oriented goal. *p < 0.05, **p < 0.01, ***p < 0.001.

## Discussion

This study aimed to explore the relationship between team behaviors, achievement goals, and competitive anxiety. Specifically, it aimed to test the mediating role of achievement goals (task-oriented and ego-oriented) in the relationship between team behaviors and competitive anxiety. The results of this study found that perceptions of controlling coaching behavior positively predicted somatic and state anxiety, and negatively predicted self-confidence. Team cohesion positively predicted self-confidence and negatively predicted state anxiety. Additionally, ego-oriented goals positively predicted competitive anxiety, while task-oriented goals negatively predicted competitive anxiety. Although several mediating pathways were not significant, overall, team behaviors positively predicted competitive anxiety through the mediating role of ego-oriented goals and negatively predicted competitive anxiety through the mediating role of task-oriented goals.

Our findings suggested a significant positive correlation between coaches’ controlling behavior and competitive anxiety among team-handball players. This implies that higher levels of coach control may lead to increased competitive anxiety, which is consistent with previous studies. Ramis et al. (2017) found that coaches’ controlling style could result in increased competitive anxiety, including somatic anxiety, worry, and concentration disruption, among athletes participating in various team sports. Similarly, Cho et al. (2019) demonstrated that controlling coaching behavior increased competitive trait anxiety among collegiate athletes. Controlling coaching behaviors through coercion, pressure, and authoritarianism establishes an atmosphere where athletes experience the pressure to perform according to the coach’s demands, leading to somatic symptoms and cognitive difficulties in focusing on the competitive situation (Cho et al., 2019). Additionally, negative emotions triggered by controlling coaching behavior could contribute to a decline in athletes’ self-confidence levels, a finding corroborated by the results of this study. This aligns with the research by Pesidas and Serrano (2023), which also supported the notion that detrimental controlling coaching behaviors could undermine the self-confidence of athletes. However, it’s crucial to acknowledge the study by (Vealey et al., 1998), which did not find a significant predictive effect of perceived coaching behavior on competitive anxiety. The primary reason for this disparity is attributed to the use of a comprehensive scale that did not differentiate between controlling and supportive coaching behaviors, potentially accounting for the differences in results. Therefore, for coaches, they should reduce controlling behaviors and appropriately increase supportive behaviors to alleviate athletes’ competitive anxiety and enhance their athletic performance.

This study also demonstrated that team cohesion had a significant predictive effect on improving athlete self-confidence and mitigating cognitive anxiety. This aligns with previous research (Prapavessis and Carron, 1996; Haddera, 2015), which indicated that athletes with higher team cohesion levels often experience lower levels of competitive anxiety and possess higher levels of self-confidence. Team cohesion plays a pivotal role in cultivating a positive psychological environment by offering emotional support, bolstering collective efficacy through a shared commitment to achieving team goals, fostering a positive and collaborative atmosphere, and building a trusting internal environment (Carron et al., 2002; Ramzaninezhad et al., 2009). Consequently, this contributes to the strengthening of collective identity, ultimately boosting athlete self-confidence and alleviating cognitive anxiety. However, Haddera (2015) found no significant associations between team cohesion and competitive anxiety among female basketball players. This may be attributed to factors such as societal expectations, cultural attitudes towards women in sports, and gender-specific social support, potentially weakening the predictive impact of team cohesion on competitive anxiety. Therefore, enhancing team cohesion may contribute to creating a supportive and favorable environment, thus potentially bolstering athletes’ self-confidence and reducing competitive anxiety. However, it’s important to note that the relationship between team cohesion and competitive anxiety may vary across different contexts and populations.

Interestingly, the study found that overall, task-oriented goals played a negative mediating role between team behavior and competitive anxiety, while ego-directed goals played a positive mediating role between team behavior and competitive anxiety. This means that task-oriented goals may weaken the positive relationship between team cohesion and competitive anxiety, thereby helping to reduce competitive anxiety among team athletes. Conversely, ego-oriented goals may strengthen this relationship, thereby increasing the level of competitive anxiety among team athletes. This finding aligns with the research by Duica et al. (2014), who found that ego-oriented goals significantly positively predicted cognitive and somatic anxiety in elite team-handball and team-volleyball athletes, whereas task-oriented goals had a significant negative predictive effect on cognitive and somatic anxiety. Typically, team athletes with a task-oriented focus prioritize understanding, gaining knowledge, and improving their competence and ability, which helps reduce the impact of external adverse factors (e.g., negative coaching behavior and excessive public expectations) on competitive anxiety (Brdar et al., 2006). In contrast, ego-oriented athletes prioritize positive evaluations from others, rendering them more susceptible to external influences (Tian et al., 2017). This heightened susceptibility exacerbates the state of competitive anxiety in athletes, as evidenced in the context of our study. Similarly, the study by Abrahamsen et al. (2008) also found team-handball players with task-oriented goals exhibit lower levels of competitive anxiety. However, it is noteworthy that the findings of this study regarding the predicting effect of team behaviors on achievement goals are inconsistent with the findings with Syrmpas and Bekiari (2018). The study by Syrmpas and Bekiari (2018) suggested a closer association between negative coaching behavior and ego-oriented goals compared to task-oriented goals, in contrast to the results of this study. This may be attributed to differences in sample characteristics (e.g., age, skill level, and cultural background). Therefore, it seems that adjusting coaching behavior to regulate the goal orientation of athletes is not an effective method. It is recommended to explore alternative approaches, such as mindfulness or meditation training, to foster a task-oriented focus among athletes and thereby alleviate competitive anxiety. Finally, for coaches, practitioners, and sports psychologists, it is advised to take more specific measures, such as integrating goal-setting strategies or mindfulness interventions to help athletes cultivate task-oriented goals, thus better managing competitive anxiety.

Although our study provides substantial insights into the relationship between team behavior, achievement goals, and competitive anxiety, it is important to acknowledge certain limitations. Firstly, a cross-sectional design was applied in this research. However, the cross-sectional design is limited in providing a clear depiction of the temporal changes in variables and causal relationships. This limitation may hinder the understanding of the dynamic relationships among team behaviors, achievement goals, and competitive anxiety. Future research should employ longitudinal designs to track changes in these variables over time and ascertain causal relationships more accurately. Secondly, the use of convenience sampling in this study limited the generalizability. Convenience sampling often fails to represent the target population adequately, potentially compromising the broader applicability of the results. Future studies should employ more rigorous probability sampling techniques to ensure a more representative sample and enhance the external validity of the findings. Thirdly, given the limited size of the sample and its diversity in terms of age and sport levels, caution is necessary in interpreting the results. The findings may be more applicable to adolescents and amateur or sub-elite populations. Fourthly, the data for this study was collected through self-report methods, particularly regarding perceptions of controlling coaching behavior. Despite using anonymous questionnaires, response biases such as social desirability bias may have influenced the results. This could have resulted in an incomplete portrayal of the true sentiments and experiences of team-handball players. To mitigate this issue, future research could consider incorporating objective measurement methods, such as behavioral observation or physiological measures, to provide more comprehensive and objective data.

## Conclusion

The results supported the notion that the perceptions of controlling coaching behavior, team cohesion, and achievement goals were of importance to understanding competitive anxiety in team-handball players. Greater levels of controlling coaching behavior may predict higher levels of athletic anxiety, while increased team cohesion may predict lower levels of competitive anxiety. Additionally, task-oriented goals negatively mediate the relationship between team behavior and competitive anxiety, whereas ego-directed goals positively mediate this relationship. Hence, enhancing team cohesion, reducing controlling coaching behavior, and emphasizing task-oriented goals appear to be effective strategies for alleviating competitive anxiety. However, to ensure the credibility of these conclusions, intervention studies are needed for further validation.

## Data availability statement

The original contributions presented in the study are included in the article/Supplementary material, further inquiries can be directed to the corresponding author.

## Ethics statement

The studies involving humans were approved by Ludong University Ethics Committee. The studies were conducted in accordance with the local legislation and institutional requirements. The participants provided their written informed consent to participate in this study.

## Author contributions

XW: Writing – original draft, Methodology, Formal analysis, Data curation, Conceptualization. ZS: Writing – review & editing, Supervision. LY: Conceptualization, Writing – original editing, Data curation. DepD: Writing – review & editing, Project administration, Methodology, Funding acquisition. DelD: Writing – original draft, Methodology, Data curation.
